# Relationship between coronary flow reserve and coronary calcium burden in patients with suspected coronary artery disease: A retrospective analysis

**DOI:** 10.1097/MD.0000000000051289

**Published:** 2026-10-30

**Authors:** Lingling Zhang, Fan Wu

**Affiliations:** a Department of Radiology, Jingxian Hospital, Anhui 242500, China.

**Keywords:** coronary artery calcium, coronary atherosclerosis, coronary blood flow, coronary flow reserve, coronary microvascular dysfunction, myocardial perfusion imaging

## Abstract

Coronary artery calcium (CAC) measures atherosclerotic burden, while coronary flow reserve (CFR) measures coronary vasodilator function. The study aimed to assess the relationship between CAC burden and CFR and to define clinical and imaging factors independently associated with compromised CFR. This retrospective observational study included 467 patients aged 16 to 93 years who underwent clinically indicated CAC assessment and quantitative ^82Rb PET/computed tomography myocardial perfusion imaging with pharmacological vasodilator stress between January 2024 and June 2026. CAC was categorized as 0, 1 to 99, 100 to 399, or ≥400 Agatston units. CFR was calculated as stress-to-rest myocardial blood flow, with CFR < 2.0 defined as impaired. Correlation, multivariable linear regression, and multivariable logistic regression analyses were performed. Mean age was 61.8 ± 13.7 years, with 59.1% male participants; 39.6% had impaired CFR. Mean CFR decreased progressively from 2.43 ± 0.62 in patients with CAC = 0 to 1.57 ± 0.47 with CAC ≥ 400 (*P* for trend < .001), while impaired CFR increased from 25.9% to 67.1%. CFR was inversely correlated with CAC (Spearman *r* = −0.43, *P* < .001). After adjustment, CAC 100 to 399 and ≥400 remained independently associated with impaired CFR (adjusted OR 2.41, 95% confidence interval 1.35 to 4.30 and OR 3.28, 95% confidence interval 1.72 to 6.25, respectively). Age, diabetes, and abnormal myocardial perfusion were also independently associated with impaired CFR. CAC was independently associated with lower CFR and a higher prevalence of impaired CFR. This association remained after multivariable adjustment, and also on sensitivity analysis, indicating that these 2 approaches to coronary assessment complement each other. The results of this study are presented as associations, due to its retrospective cross sectional design.

## 1. Introduction

Coronary artery disease (CAD) is a major cause of cardiovascular morbidity and mortality worldwide. Most myocardial ischaemias and adverse cardiovascular outcomes are due to the chronic evolution of coronary atherosclerosis, and characterization of total atherosclerotic burden is a critical part of current cardiovascular risk management. While most diagnostic methods have focused on obstructive lesions, recent strategies have added both structural plaque burden and coronary vascular function to enhance risk stratification. Anatomical and functional imaging can thus help identify patients at high cardiovascular risk before a clinical event.^[1]^ Early detection of subclinical or advanced CAD can also contribute to timely preventive measures, especially in patients with suspected CAD.^[2]^

Coronary artery calcium (CAC) is a well-known noninvasive index of the burden of coronary atherosclerotic disease. Non-contrast cardiac computed tomography (CT), measured by the Agatston method, quantitatively assesses calcified coronary plaque and is widely used for cardiovascular risk assessment. CAC scores are typically divided into 4 categories: 0, 1 to 99, 100 to 399, and ≥400 Agatston units (AUs), corresponding to no calcification, mild calcification, moderate calcification, and severe calcification, respectively.^[3]^ A CAC score of zero is typically considered good, but does not rule out the presence of noncalcified plaque and/or abnormalities in coronary vascular function. However, higher CAC correlates with higher anatomical complexity and worse functional parameters in hybrid cardiac imaging.^[4]^

Coronary flow reserve (CFR) gives complementary data by evaluating the functional capacity of the coronary circulation. CFR measures the ratio of stress to resting myocardial blood flow (MBF) and reflects integrated epicardial coronary artery and coronary microcirculatory vasodilator capacity. A reduced CFR (typically <2.0) can be due to CAD (obstruction), diffuse atherosclerosis, endothelial dysfunction, or coronary microvascular dysfunction.^[5]^ Quantitative analysis of CFR can therefore detect clinically relevant abnormalities that are not seen by conventional perfusion imaging and is associated with a higher cardiovascular risk.^[6]^ Importantly, reduced coronary vasodilator function is a predictor of poor clinical outcomes even after accounting for the severity of epicardial coronary stenosis.^[7]^

There is growing evidence that there is an important relationship between coronary calcification and impaired coronary vasodilator function. There has been a progressive decline in hyperemic MBF and CFR with increasing CAC burden.^[8]^ Hybrid positron emission tomography/computed tomography (PET/CT) imaging can potentially provide a more comprehensive characterization of coronary disease by combining CAC with quantitative MBF.^[9]^ Furthermore, abnormal CFR has predictive value even among those with normal myocardial perfusion imaging (MPI).^[10]^ More recently, it has been shown that impaired CFR (also termed myocardial flow reserve) can occur in people who have no detectable coronary calcium (or a CAC score of zero),^[11]^ which means that the presence of coronary calcium does not automatically imply that there is any problem with the coronary vessels.

Despite these results, the independent association of CAC burden with impaired CFR remains poorly understood. Longitudinal imaging studies have also indicated that progression of structural coronary calcification and changes in myocardial perfusion are not necessarily correlated.^[12]^ Hence, the present study aimed to explore the relationship between CAC burden and CFR among patients suspected of having CAD. Secondary objectives included determining the number of patients with impaired CFR by CAC category (0, 1 to 99, 100 to 399, ≥400 AUs) and identifying independent clinical and imaging predictors of impaired coronary vasodilator function.

## 2. Methodology

### 2.1. Study design and setting

This retrospective observational study explored the association between CFR and CAC burden in patients referred for suspected CAD. The study was conducted on routinely collected clinical, imaging and diagnostic data of patients undergoing clinically indicated coronary calcium assessment and quantitative MPI at a tertiary cardiovascular center. The study period extended from January 1, 2024, to June 30, 2026. The study was designed under the methodological principles of the recent studies assessing the complementary value of coronary atherosclerotic burden, myocardial perfusion and quantitative coronary flow measures.

### 2.2. Study population and participant selection

Patients were referred for noninvasive cardiovascular evaluation for suspected CAD between 16 and 93 years of age (the study population). A retrospective review of electronic medical records, image databases, and nuclear cardiology reports was conducted. Patients with quantitative MBF during rest and stress were eligible, provided they had undergone coronary calcium assessment. They had had a MPI scan during the study period.

The total number of patient records identified was 612. Patient records for 584 unique patients were screened after duplicate or repeat examinations were removed. In total, 117 patients were excluded during detailed record review due to incomplete or technically suboptimal coronary calcification imaging, missing or unreliable quantitative coronary blood flow measurements, missing clinical data needed for the planned analyses, or imaging studies deemed unsuitable for quantitative interpretation. The final analytical sample consisted of 467 patients.

A retrospective study flow diagram was used to document the participant selection process. The flow diagram showed the number of records initially identified, the number of records that were excluded after being duplicated, the number of patients who were screened, the number of records excluded due to reasons, and the number of patients included in analysis. Because this was a retrospective study, no direct patient recruitment or interventions were conducted.

### 2.3. Eligibility criteria

Patients included had a defined study period of 16 to 93 years. They had symptoms or clinical findings suggestive of CAD, had coronary calcium assessment and quantitative myocardial perfusion imaging (QMPI) available during the defined time frame, and had interpretable measurements of resting and stress MBF. Inclusion criteria included complete information on primary exposure (CAC burden) and primary functional outcome (CFR).

Patients were excluded if they had repeat exams, incomplete or missing imaging information, technically inadequate attenuation-corrected CT scans, nondiagnostic MPI exams, unreliable MBF quantification, or extensive missing data regarding important covariates. If there was more than 1 eligible examination for the same patient, the first completed examination within the study period was kept to avoid multiple observations from the same person.

There was no lower age limit placed after the data were extracted; thus, all patients who were seen consecutively for clinically indicated evaluation for possible CAD during the study period were evaluated. In the final cohort, 6 young people (age 16–17) were included. These patients were referred for cardiovascular assessment due to documented clinically suspected and/or premature coronary disease. The participants listed below (below 18 years old) were covered by the approval of the Jingxian Hospital Ethics Committee (Approval Number: 202603).

The analytical cohort excluded patients with known CAD as these patients were not the ones to be assessed in the study for suspected rather than known CAD. During the eligibility process, 18 patients with a history of previous myocardial infarction or coronary revascularization were excluded. The exclusions for these are included in the 117 patients excluded after screening and are thus represented in the participant flow diagram.

### 2.4. Clinical and demographic data collection

Demographic and clinical data were obtained from electronic medical records with a standardized data collection form. Age, sex, presenting symptoms, body mass index (BMI), hypertension, diabetes mellitus, dyslipidaemia, smoking and family history of premature cardiovascular disease, and use of relevant cardiovascular medication were recorded. Previous cardiovascular disease information and other clinically relevant comorbidities were also reviewed if available.

Age was analyzed as a continuous variable and, as appropriate, as clinically relevant age groups. Hypertension was determined with either documented clinical diagnosis or use of antihypertensive drugs or raised blood pressure reading taken to institutional standards. Diabetes mellitus was defined as having a history of diagnosis, using glucose-lowering therapy, or laboratory findings meeting established diagnostic criteria.^[13]^ Medical records were used to identify dyslipidemia based on a diagnosis, lipid medication, or an abnormal lipid profile. If enough data were provided, smoking status was defined as a current, former or never smoker.

### 2.5. CAC assessment

CAC was quantified using the Agatston scoring method.^[14]^ The total CAC score was used as the primary anatomical measure of calcified coronary atherosclerotic burden. CAC acquisition and scoring followed institutional protocols consistent with established coronary calcium-scoring recommendations.^[15]^ Non-contrast CT for calcium-scoring was obtained with a low dose, ECG-synchronized calcium-scoring acquisition at 120 kVp, and 3.0 mm reconstructed slices, on a dedicated low dose ECG-synchronized calcium-scoring acquisition, acquired on the hybrid PET/CT system (Biograph mCT, Siemens Healthineers, Germany). Calcified lesions were considered abnormal regions with an area of at least 3 contiguous pixels (area ≥1 mm^2^) and attenuation ≥130 HU. Two experienced, blinded radiologists used manufacturer-provided and validated cardiac analysis software to arrive at the Agatston score; this score was determined independently of the quantitative MBF results. If there was disagreement the final decision was based on consensus, Regular quality control checks of the quality of the images used for calcium measurement were performed prior to analysis.

CAC burden was divided into 4 categories for descriptive/comparative analysis: 0, 1 to 99, 100 to 399 and ≥400, AUs, represented the absence of CAC, mild CAC, moderate CAC and extensive CAC, respectively.^[15]^ Due to the right-skewed distribution of the CAC score, it was also analyzed as a continuous variable following appropriate transformation. The CAC categories were chosen to make clinically meaningful comparisons between different levels of coronary atherosclerotic burden.

### 2.6. QMPI and CFR

QMPI was performed using ^82Rb PET/CT on a Biograph mCT PET/CT system (Siemens Healthineers, Germany). Patients were advised to not consume any food or drinks for about 4 hours prior to the imaging exam, and to not drink anything containing caffeine for at least 12 hours before the exam. Rest imaging was done first, followed by pharmacological vasodilator stress imaging with adenosine (intravenous infusion at 140 µg/kg/min for 6 minutes). Continuous electrocardiographic monitoring was done during the stress imaging. Dynamic list-mode PET data were collected for 6 minutes beginning with each injection of ^82Rb and static clinical data were collected as usual. PET data were reconstructed with ordered subset iterative reconstruction (ITER) algorithm with CT-based attenuation correction and with routine post-reconstructed filtering in the institution.

Cardiac PET analysis software validated under a compartmental tracer-kinetic model was used to quantify global LV resting and stress MBF (mL/g/minutes) based on commercially available PET analysis software.^[16]^ Quality control involved patient motion, PET/CT misregistration, left-ventricular myocardial contours and the arterial input function obtained from blood-pool time–activity curve. If technically possible, PET/CT misregistration was corrected manually. The haemodynamic response to adenosine was also discussed to evaluate the effectiveness of the pharmacological vasodilator. Reliability of the measurements was judged by the quality of the motion data, which included the presence of substantial uncorrectable motion or PET/CT misregistration; quality of the arterial input function; technical incompleteness of the acquisition; and physiologic implausibility of the measurements (Fig. 1); and such measurements were excluded from the final analysis. The 2 nuclear medicine physicians who were blinded to the results of the CAC reviewed the quantitative PET studies.

**Figure 1.**
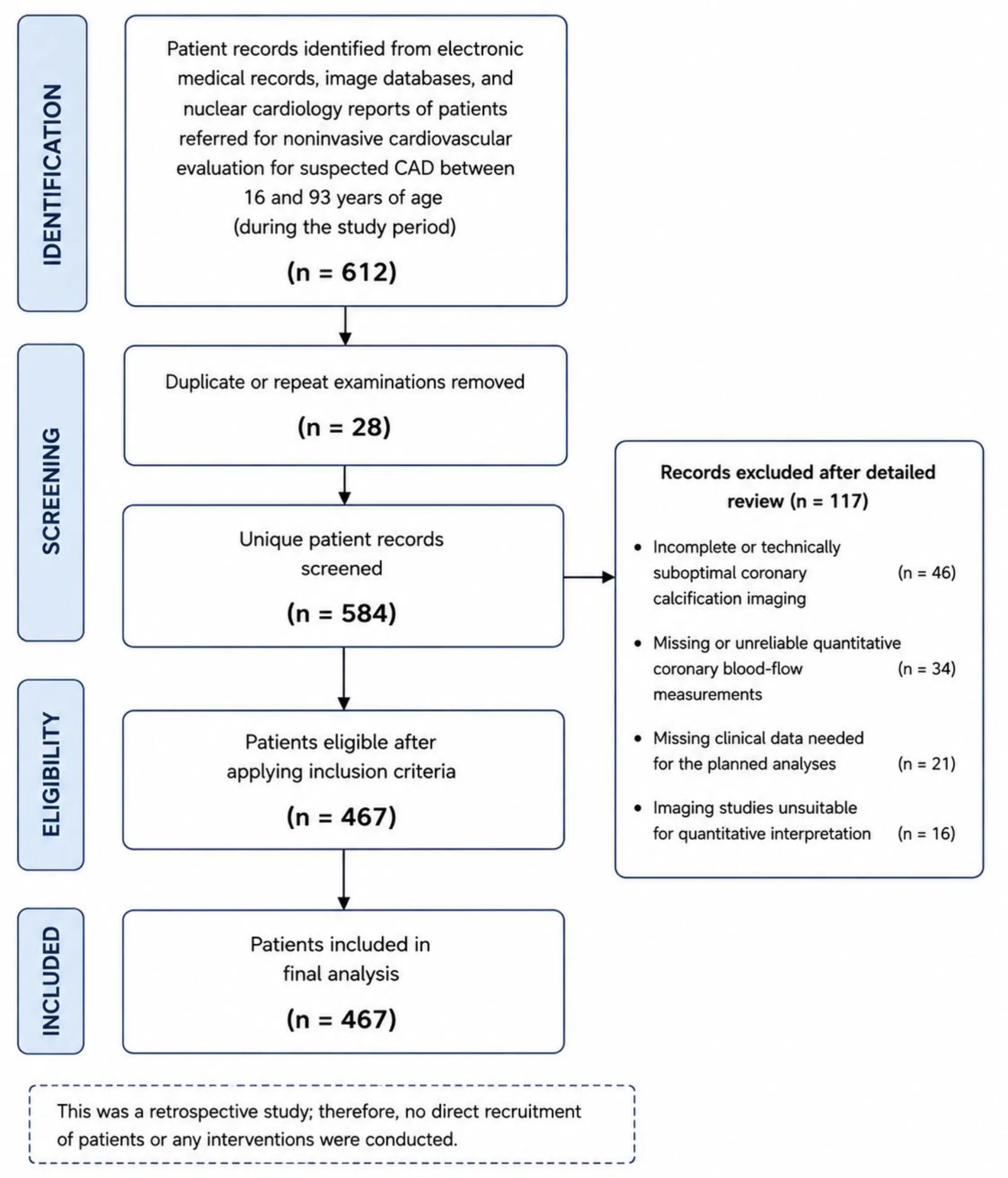
Participant selection flow diagram.

CFR was calculated as the ratio of stress MBF to resting MBF^[16]^:


CFR=Stress myocardial blood flow/Resting myocardial blood flow

CFR was evaluated as a continuous variable to assess its association with CAC burden. Impaired CFR was prespecified as <2.0, whereas CFR ≥2.0 was considered preserved. A CFR threshold of <2.0 is commonly used to identify reduced coronary vasodilator capacity and may reflect the effects of epicardial CAD, diffuse coronary atherosclerosis, or coronary microvascular dysfunction.^[16,17]^

CFR was interpreted as an integrated measure of coronary vasodilator function because it reflects the combined influence of epicardial coronary and coronary microvascular function and does not, by itself, distinguish between obstructive epicardial disease and microvascular dysfunction.

### 2.7. Myocardial perfusion and additional imaging variables

Results of MPI were obtained from final clinical imaging reports. Perfusion was considered normal or abnormal based on the presence of reversible ischemia, fixed perfusion defects, or other clinically important perfusion defects. If available, quantitative or semiquantitative perfusion indices were noted and included in adjusted analyses.

The correlation between CAC burden and CFR were analyzed separately and in relation to myocardial perfusion results. This enabled assessment of whether coronary calcium burden was related to impaired coronary flow in the absence of visually or quantitatively detected perfusion abnormalities.

No invasive coronary angiography or coronary CT angiography was required for inclusion and only performed where clinically indicated. Angiographic data within 90 days of perfusion imaging were available in (142 [30.4%]) patients, 87 (61.3%) of whom had obstructive CAD (the left main artery ≥50%, or a major epicardial artery ≥70%). These data were only available for a subset clinically selected, therefore obstructive CAD was not included as a covariate in the primary model which is recognized as a limitation.

### 2.8. Operational definitions of key variables

Coronary calcium burden, represented by the CAC score, was used as the primary exposure variable and divided into 4 categories: 0, 1 to 99, 100 to 399, and ≥400. CFR, which was defined as the ratio of stress to resting MBF, was the primary outcome variable. Impaired CFR was defined in this analysis as CFR <2.0.

Suspected CAD was defined as the presence of symptoms, clinical risk factors, abnormal cardiovascular findings, or physician concern warranting noninvasive evaluation for possible CAD, consistent with contemporary chest pain evaluation guidance.^[18]^ A coronary calcium burden of ≥400 was considered high. Final imaging interpretation of “normal myocardial perfusion” was defined as the absence of clinically significant inducible and/or fixed perfusion abnormality. In contrast, “abnormal myocardial perfusion” was defined as the presence of a clinically relevant perfusion defect.

The primary association of interest was between the CAC burden and the CFR. Additionally, the study examined whether the CAC burden remained significantly associated with CFR after controlling for demographic characteristics, cardiovascular risk factors, and myocardial perfusion results.

### 2.9. Data quality and management of missing data

Clinical and imaging data were independently obtained from the clinical and imaging records and subsequently reviewed for their completeness, internal consistency and possible entry errors. Prior to the final analysis, duplicate records were deleted. Technically less reliable imaging techniques with quantitative measurements were not included to minimize the possibility of measurement-related bias.

Prior to statistical analysis, extent and pattern of missing data were determined. Individuals without either the main exposure or the main result were discarded from the final analytical cohort. Complete-case analysis was used when covariate missingness was small and the probability that missing values would substantially affect the estimates was low. Multivariable models excluded variables with high rates of missing data. Information on the number of patients included in each analysis was provided when it differed from the total number of patients in the study. At screening, 21 patients were excluded from the present cohort due to missing clinical data required to undergo the planned analyses (Fig. 1). All patients in final analytical cohort (467) had complete data for exposure (CAC) and outcome (CFR) and no imputation was necessary for the multivariable models since all the covariates were complete. All multivariable models (Tables 4–5 and 7) were therefore fitted in the same 467 patients (185 patients with impaired CFR); the number of patients included in the subgroup analyses is reported in Table 7.

**Table 4:** Association between coronary calcium burden and impaired coronary flow reserve.

CAC category	Impaired CFR, n/N (%)	Unadjusted OR (95% CI)	*P* value	Adjusted OR* (95% CI)	*P* value
CAC = 0	50/193 (25.9)	Reference	–	Reference	–
CAC 1–99	40/118 (33.9)	1.47 (0.89–2.43)	.132	1.29 (0.76–2.19)	.344
CAC 100–399	48/86 (55.8)	3.62 (2.12–6.18)	<.001	2.41 (1.35–4.30)	.003
CAC ≥ 400	47/70 (67.1)	5.84 (3.23–10.55)	<.001	3.28 (1.72–6.25)	<.001

**Table 5:** Clinical and imaging predictors of impaired coronary flow reserve.

Variable	Univariable OR (95% CI)	*P* value	Adjusted OR (95% CI)	*P* value
Age, per 10-yr increase	1.46 (1.25–1.71)	<.001	1.27 (1.06–1.53)	.010
Male sex	1.39 (0.96–2.01)	.081	1.17 (0.77–1.79)	.453
Body mass index, per 5 kg/m^2^	1.13 (0.94–1.36)	.187	1.08 (0.88–1.32)	.468
Hypertension	2.12 (1.47–3.07)	<.001	1.36 (0.89–2.09)	.157
Diabetes mellitus	2.37 (1.60–3.51)	<.001	1.71 (1.10–2.66)	.017
Dyslipidemia	1.69 (1.17–2.43)	.005	1.22 (0.80–1.87)	.354
Current/former smoking	1.29 (0.85–1.96)	.229	1.11 (0.70–1.76)	.666
Chronic kidney disease	2.52 (1.34–4.73)	.004	1.64 (0.82–3.29)	.164
CAC 1–99	1.47 (0.89–2.43)	.132	1.29 (0.76–2.19)	.344
CAC 100–399	3.62 (2.12–6.18)	<.001	2.41 (1.35–4.30)	.003
CAC ≥ 400	5.84 (3.23–10.55)	<.001	3.28 (1.72–6.25)	<.001
Abnormal myocardial perfusion	3.05 (2.05–4.54)	<.001	2.14 (1.37–3.35)	.001

**Table 7:** Sensitivity and subgroup analyses of the association between CAC burden and impaired CFR.

Analysis	Effect estimate (95% CI)	*P* value
Log-transformed CAC and continuous CFR (n = 467)	Adjusted β = −0.21 (−0.28–− 0.14)	<.001
CAC ≥ 400 and impaired CFR in the overall cohort (n = 467)	Adjusted OR = 3.28 (1.72–6.25)	<.001
CAC ≥ 400 and impaired CFR among patients with normal perfusion (n = 336)	Adjusted OR = 2.74 (1.28–5.87)	.009
Moderate/high CAC and impaired CFR among patients without diabetes (n = 326)	Adjusted OR = 2.19 (1.28–3.74)	.004
Moderate/high CAC and impaired CFR among patients with diabetes (n = 141)	Adjusted OR = 3.07 (1.48–6.39)	.003
CAC burden and CFR among male patients (n = 276)	Adjusted β = −0.23 (−0.33–− 0.13)	<.001
CAC burden and CFR among female patients (n = 191)	Adjusted β = −0.18 (−0.30–− 0.06)	.004

### 2.10. Data sources and measurement

Information was collected from the institution’s electronic health record, the cardiovascular imaging database, the nuclear medicine reporting system, and clinical and laboratory records. A standardized approach to data collection was used to extract the following demographic, cardiovascular risk factor, clinical history, medication, coronary calcium, myocardial perfusion, and quantitative MBF parameters. Finalized clinical reports and clinical imaging datasets were used to record imaging-derived variables when available. Definitions for variables and coding procedures were formulated prior to data extraction, and selected records were double-checked for completeness and consistency.

### 2.11. Potential sources of bias

This retrospective design could have led to selection bias, as patients were included only if they were referred for clinically indicated coronary imaging; hence, the results may not be fully applicable to the asymptomatic population or to people without suspected CAD. Information bias could have resulted from inadequate documentation, inconsistent clinical record quality, or inconsistent imaging acquisition and reporting. The impact of unmeasured residual confounding (e.g., degree of diffuse coronary atherosclerosis, microvascular dysfunction, or clinical variables not routinely reported) was not completely ruled out. Predefined eligibility criteria, uniform variable definitions, exclusion of technically unacceptable examinations, and data verification and multivariable adjustment for relevant demographic and clinical covariates minimized the potential for bias.

### 2.12. Statistical analysis

IBM SPSS Statistics for Windows, version 29.0 (IBM Corp., Armonk) was used for statistical analysis. Graphical techniques and suitable tests of normality were used to examine the distributional properties of the continuous variables. Mean ± standard deviation (SD) and median (IQR) were used for normally distributed and nonnormally distributed variables, respectively. Categorical data were presented as frequencies and percentages. One-way analysis of variance or the Kruskal–Wallis test was used to compare baseline characteristics among the CAC categories as appropriate. The chi-square test or Fisher’s exact test was used to compare categorical variables. Appropriate trend analyses were performed to assess trends in CFR for increasing CAC categories.

A correlation analysis was first applied to the association between CAC burden and CFR. Pearson correlation was used for continuous data that were approximately normally distributed, and Spearman rank correlation was used for continuous data that were nonnormally distributed. Multivariable linear regression was used to assess the independent association of continuous CFR and CAC burden adjusted for potential confounders. CAC scores were logarithmically transformed when needed to reduce skewness and improve model fit.

Factors independently associated with impaired CFR were determined using multivariable logistic regression. Impaired CFR (<2.0) was the dependent variable, and candidate independent variables were age, sex, cardiovascular risk factors, CAC burden and myocardial perfusion status. Odds ratios (ORs) with 95% confidence intervals (CIs) were presented. A single multivariable logistic regression model was used, which included age (per 10-year increase), sex, BMI (per 5 kg/m^2^), hypertension, diabetes mellitus, dyslipidaemia, smoking status, chronic kidney disease (CKD), abnormal myocardial perfusion and CAC category (entered as a 4-level categorical variable with CAC = 0 as the reference). The estimates for the CAC presented in Table 4 and estimates for all covariates presented in Table 5 are estimates from the same model. All the same covariates were used in the multivariable linear regression for CFR, which was continuous. Model performance was evaluated using adequate goodness-of-fit measures, and multicollinearity was assessed prior to model building. A two-sided *P* value < .05 was defined as statistically significant. Effect estimates were reported with 95% CIs to give information about the size and precision of the observed associations.

#### 2.12.1. Sample size and power

This was a retrospective study whereby no prior sample size calculation was done and all eligible consecutive patients who were examined during the study period were included. Two measures were used to determine the sample adequacy. One first was that the multivariable logistic regression model had 185 events (outcome events) and 12 parameters (covariates plus CAC indicator variables), or about 15 events per parameter, which is above the rule of thumb of 10 events per parameter.^[19]^ Second, to demonstrate the power of the study, a post hoc sensitivity power calculation (two-sided α = 0.05) was performed: With 193 patients in the CAC = 0 group, the study had 80% power to detect an increase in the prevalence of impaired CFR of 25.9% to 44.4% in the CAC ≥ 400 group (n = 70) and to 43.0% in the CAC 100 to 399 group (n = 86); for the continuous analysis, 467 patients provided 80% power to detect a correlation coefficient of |*r*| ≥ 0.13.

Sensitivity and Subgroup analyses. Log-transformed CAC was analyzed as a continuous variable as part of planned analyses (Section 2.5). All the subgroup analyses (myocardial perfusion status, diabetes status and sex) were exploratory. These analyses were not formally adjusted for multiple comparisons and so the results are presented as hypothesis generating due to the possibility of a type I error, and a Bonferroni corrected significance level is also reported for reference.

### 2.13. Ethical considerations

This research has been approved by the Jingxian Hospital Ethics Committee, Approval Number: 202603. Ethical principles of the Declaration of Helsinki and relevant amendments were followed in the study. No patient identifiers were added to the analytical dataset, and all results were presented in summary form. The Ethics Committee approved this retrospective study, which waived the informed consent requirement for all patients in the study cohort, including those ages 16 to 17 years.

## 3. Results

### 3.1. Participant selection and study cohort

During the study period, a total of 612 imaging records were identified. Of these, 584 were unique patient records which were screened. After careful assessment of the eligibility criteria, 117 patients were excluded due to poor or incomplete coronary calcium images, inability to obtain quantitative coronary blood flow assessment, missing clinical data, or inadequate imaging studies. Finally, 467 patients had complete and interpretable coronary calcium and quantitative coronary flow measurements, as shown in Figure 1.

The flow diagram illustrates the identification, duplicate removal, eligibility screening, exclusion, and final inclusion of patients. Of 612 initially identified imaging records, 467 patients with complete and interpretable CAC and quantitative coronary flow measurements were included in the final analysis.

### 3.2. Baseline demographic and clinical characteristics

The final study cohort included 467 patients aged 16 to 93 years, with a mean age of 61.8 ± 13.7 years; 45.8% were aged ≥65 years and 59.1% were male. Cardiometabolic risk factors were found in many of the patients, such as hypertension (57.2%), dyslipidemia (49.0%), diabetes mellitus (30.2%) and overweight or obesity (63.6%). The median CAC score was 96 (IQR, 0–382) AU, while 41.3% had a CAC score of 0. Mean resting and stress MBF were 0.91 ± 0.22 and 1.96 ± 0.67 mL/g/min, respectively, resulting in a mean CFR of 2.18 ± 0.66. Overall, 39.6% of patients had abnormal CFR (<2.0) and 28.1% had abnormal myocardial perfusion. Demographic, clinical and imaging baseline parameters are fully summarized in the Table 1.

**Table 1:** Baseline demographic, clinical, and imaging characteristics of the study cohort (N = 467).

Variable	Overall cohort
Age, yr, mean ± SD	61.8 ± 13.7
Age range, yr	16–93
Age≥65 yr, n (%)	214 (45.8)
Male sex, n (%)	276 (59.1)
Female sex, n (%)	191 (40.9)
Body mass index, kg/m^2^, mean ± SD	27.4 ± 4.6
Overweight or obesity, n (%)	297 (63.6)
Hypertension, n (%)	267 (57.2)
Diabetes mellitus, n (%)	141 (30.2)
Dyslipidemia, n (%)	229 (49.0)
Current or former smoking, n (%)	111 (23.8)
Family history of premature cardiovascular disease, n (%)	82 (17.6)
Chronic kidney disease, n (%)	43 (9.2)
Previous cerebrovascular disease, n (%)	31 (6.6)
Typical angina, n (%)	143 (30.6)
Atypical chest pain, n (%)	176 (37.7)
Dyspnea as the principal symptom, n (%)	94 (20.1)
Other or nonspecific symptoms, n (%)	54 (11.6)
Statin therapy, n (%)	218 (46.7)
Antihypertensive therapy, n (%)	249 (53.3)
Glucose-lowering therapy, n (%)	119 (25.5)
CAC score, AU, median (IQR)	96 (0–382)
CAC score = 0, n (%)	193 (41.3)
CAC score > 0, n (%)	274 (58.7)
Rest myocardial blood flow, mL/g/min, mean ± SD	0.91 ± 0.22
Stress myocardial blood flow, mL/g/min, mean ± SD	1.96 ± 0.67
Coronary flow reserve, mean ± SD	2.18 ± 0.66
Impaired CFR (<2.0), n (%)	185 (39.6)
Abnormal myocardial perfusion, n (%)	131 (28.1)
Inducible ischemia, n (%)	96 (20.6)
Predominantly fixed perfusion defect, n (%)	35 (7.5)

Data are presented as mean ± standard deviation (SD), median with interquartile range (IQR), or frequency with percentage.

AU = Agatston units, CAC = coronary artery calcium, CFR = coronary flow reserve.

Data are presented as mean ± SD, median with interquartile range (IQR), or frequency with percentage. CAC, AU, CFR.

### 3.3. Distribution of coronary calcium burden

Significant differences in demographics, cardiovascular risk factors and imaging features were seen between the 4 categories of CAC. Patients had a progressive increase in mean age from 53.7 ± 13.5 years for those with CAC = 0 to 72.1 ± 8.7 years for those with CAC ≥ 400 (*P* < .001), and the prevalence of hypertension, diabetes, dyslipidemia, and CKD also increased as the calcium burden increased. There was a significant difference in abnormal myocardial perfusion between the CAC = 0 group and the CAC ≥ 400 group (16.6% vs 48.6%, *P* < .001). Importantly, there was a progressive decrease in mean CFR (from 2.43 ± 0.62 to 1.57 ± 0.47, both *P* < .001) and a corresponding progressive increase in the proportion of impaired CFR (from 25.9% to 67.1%, both *P* < .001) across the CAC categories. Details of such comparisons are given in Table 2.

**Table 2:** Clinical and imaging characteristics according to coronary artery calcium burden.

Variable	CAC = 0 (n = 193)	CAC 1–99 (n = 118)	CAC 100–399 (n = 86)	CAC ≥ 400 (n = 70)	*P*-value
Age, yr, mean ± SD	53.7 ± 13.5	62.4 ± 11.2	68.0 ± 9.4	72.1 ± 8.7	<.001
Male sex, n (%)	94 (48.7)	73 (61.9)	62 (72.1)	47 (67.1)	<.001
Body mass index, kg/m^2^	26.9 ± 4.5	27.5 ± 4.7	28.0 ± 4.4	27.8 ± 4.6	.118
Hypertension, n (%)	84 (43.5)	71 (60.2)	64 (74.4)	48 (68.6)	<.001
Diabetes mellitus, n (%)	39 (20.2)	34 (28.8)	38 (44.2)	30 (42.9)	<.001
Dyslipidemia, n (%)	71 (36.8)	59 (50.0)	55 (64.0)	44 (62.9)	<.001
Current/former smoking, n (%)	37 (19.2)	29 (24.6)	25 (29.1)	20 (28.6)	.102
Chronic kidney disease, n (%)	8 (4.1)	10 (8.5)	13 (15.1)	12 (17.1)	<.001
Typical angina, n (%)	49 (25.4)	36 (30.5)	33 (38.4)	25 (35.7)	.041
Statin therapy, n (%)	58 (30.1)	58 (49.2)	61 (70.9)	41 (58.6)	<.001
Abnormal myocardial perfusion, n (%)	32 (16.6)	29 (24.6)	36 (41.9)	34 (48.6)	<.001
Inducible ischemia, n (%)	22 (11.4)	20 (16.9)	29 (33.7)	25 (35.7)	<.001
Rest myocardial blood flow, mL/g/min	0.88 ± 0.20	0.90 ± 0.21	0.95 ± 0.23	1.00 ± 0.25	.002
Stress myocardial blood flow, mL/g/min	2.14 ± 0.66	1.98 ± 0.61	1.76 ± 0.57	1.54 ± 0.52	<.001
CFR, mean ± SD	2.43 ± 0.62	2.20 ± 0.57	1.88 ± 0.54	1.57 ± 0.47	<.001
Impaired CFR, n (%)	50 (25.9)	40 (33.9)	48 (55.8)	47 (67.1)	<.001

CAC categories represent absent, mild, moderate, and extensive coronary calcification. Continuous variables were compared using one-way analysis of variance or the Kruskal–Wallis test, as appropriate. Categorical variables were compared using the chi-square test. CFR.

A clear gradient was observed across CAC categories. Higher calcium burden was associated with older age, a greater prevalence of cardiometabolic risk factors, more frequent abnormal myocardial perfusion, lower stress MBF, and progressively reduced CFR. The prevalence of impaired CFR increased from 25.9% among patients with CAC = 0 to 67.1% among those with CAC ≥ 400.

### 3.4. CFR across categories of coronary calcium burden

A consistent relationship was found between increasing CAC burden and quantitative coronary flow measurements of a decline in coronary vasodilator function. Resting MBF increased modestly from 0.88 ± 0.20 mL/g/minutes in patients with CAC = 0 to 1.00 ± 0.25 mL/g/minutes in those with CAC ≥ 400 (*P* for trend = .001), whereas stress MBF decreased from 2.14 ± 0.66 to 1.54 ± 0.52 mL/g/minutes (*P* for trend <.001). Consequently, mean CFR declined from 2.43 ± 0.62 in the absence of CAC to 1.57 ± 0.47 in patients with CAC ≥ 400 (*P* for trend < .001). The percent of patients with low CFR rose as well from 25.9% to 67.1%. Results of the quantitative flow are summarized in Table 3.

**Table 3:** Quantitative coronary flow measurements according to coronary calcium category.

Quantitative parameter	CAC = 0	CAC 1–99	CAC 100–399	CAC ≥ 400	*P* for trend
Rest myocardial blood flow, mL/g/min	0.88 ± 0.20	0.90 ± 0.21	0.95 ± 0.23	1.00 ± 0.25	.001
Stress myocardial blood flow, mL/g/min	2.14 ± 0.66	1.98 ± 0.61	1.76 ± 0.57	1.54 ± 0.52	<.001
CFR, mean ± SD	2.43 ± 0.62	2.20 ± 0.57	1.88 ± 0.54	1.57 ± 0.47	<.001
CFR, median (IQR)	2.35 (2.00–2.81)	2.14 (1.82–2.52)	1.80 (1.48–2.19)	1.51 (1.22–1.87)	<.001
Preserved CFR (≥2.0), n (%)	143 (74.1)	78 (66.1)	38 (44.2)	23 (32.9)	<.001
Impaired CFR (<2.0), n (%)	50 (25.9)	40 (33.9)	48 (55.8)	47 (67.1)	<.001

Data are presented as mean ± SD, median (IQR), or n (%). CFR was calculated as stress MBF divided by resting MBF. A CFR < 2.0 was classified as impaired.

This progressive decrease in CFR was also seen graphically for the different categories of coronary calcium. The CFR was inversely related to the calcium burden, with a mean CFR of 2.43 in patients with CAC = 0 and decreasing to 2.20, 1.88, and 1.57 in those with CAC 1 to 99, 100 to 399, and ≥400, respectively. This progressive decrease is presented in Figure 2, where the CIs (95%) are shown.

**Figure 2.**
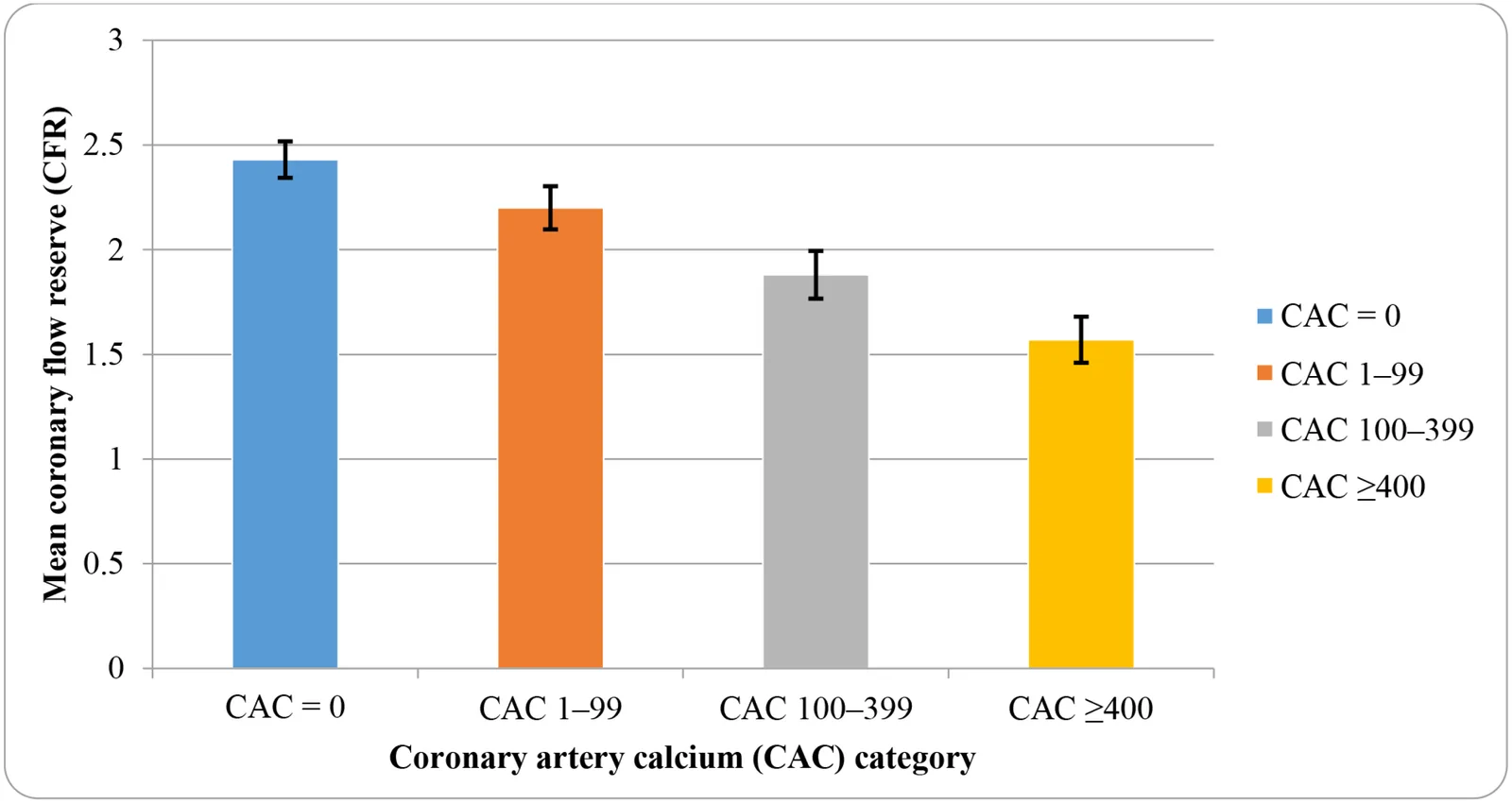
Coronary flow reserve according to coronary artery calcium burden. CAC = coronary artery calcium.

Bars show mean CFR values across the 4 CAC categories (CAC = 0, n = 193; CAC 1 to 99, n = 118; CAC 100 to 399, n = 86; CAC ≥ 400, n = 70). A progressive reduction in CFR is observed with increasing CAC burden, with the lowest mean CFR among patients with CAC ≥ 400. Error bars represent 95% CIs of the mean (CAC = 0: 2.34–2.52; CAC 1–99: 2.10–2.30; CAC 100–399: 1.77–1.99; CAC ≥ 400: 1.46–1.68).

### 3.5. Relationship between continuous coronary calcium burden and CFR

A significant inverse association between coronary calcium burden and CFR was found when the coronary calcium burden was used as a continuous variable. Logarithmized CAC values were used due to the right-skewed distribution, and higher CAC scores were related to lower CFR (Spearman *r* = −0.43; *P* < .001). A negative correlation was also seen between CAC burden and stress MBF (*r* = −0.39, *P* < .001), and a positive correlation was observed between CAC burden and resting myocardial blood flow (*R* = 0.17, *P* < .001). The cross-sectional relationship between CAC burden and CFR is shown in Figure 3.

**Figure 3.**
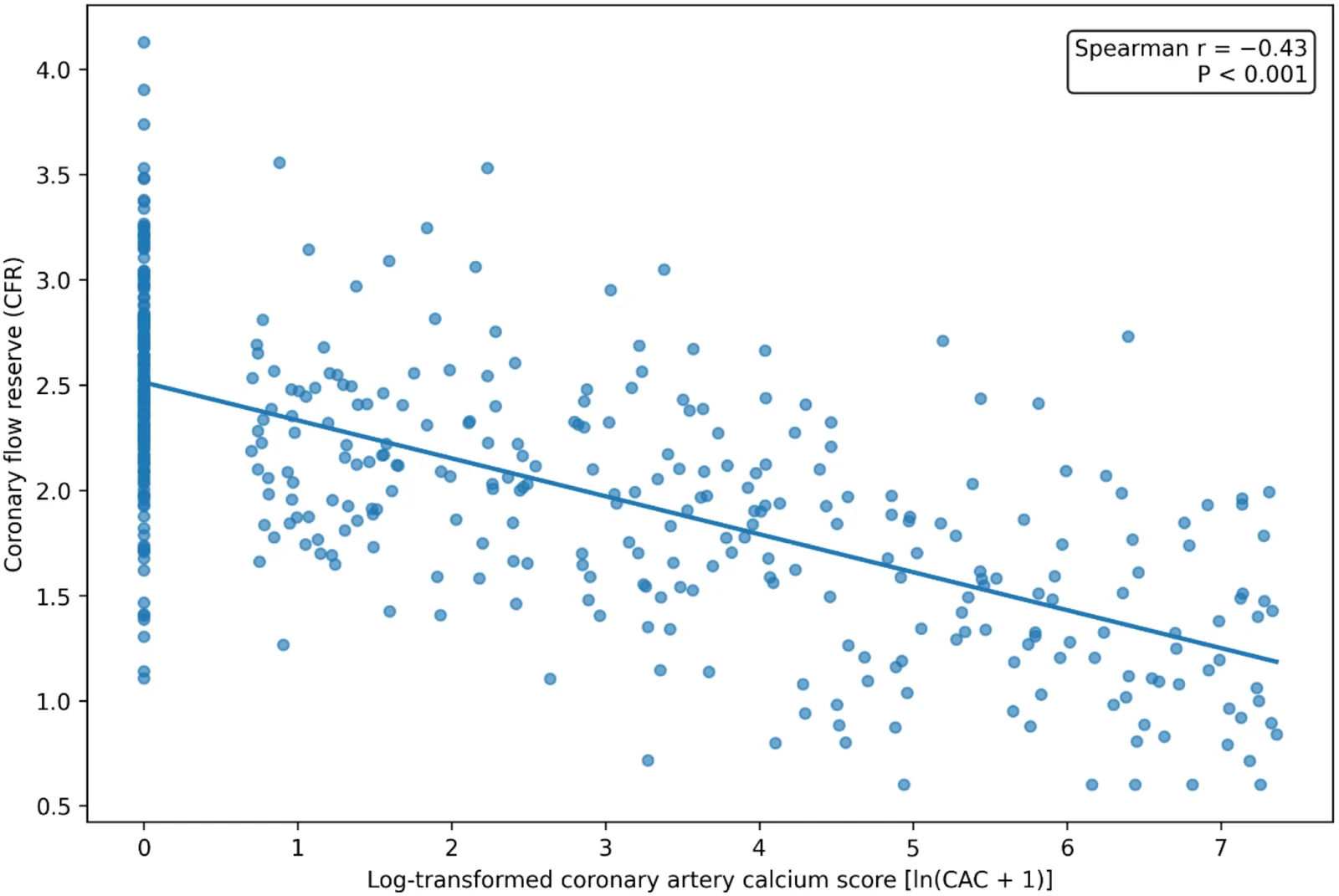
Association between coronary calcium burden and coronary flow reserve.

Scatter plot of the log-transformed CAC score [ln(CAC + 1)] (x-axis) against CFR (y-axis). Each point represents 1 patient (N = 467). The solid line is the fitted least-squares regression line and illustrates the inverse association between CAC burden and CFR (Spearman *r* = −0.43; *P* < .001).

### 3.6. Coronary calcium burden and impaired CFR

The prevalence and adjusted odds of impaired CFR were higher in higher CAC categories. Compared with patients with CAC = 0, those with CAC 100 to 399 had more than twice the adjusted odds of impaired CFR (adjusted OR 2.41, 95% CI 1.35–4.30; *P* = .003), while patients with CAC ≥ 400 had more than threefold higher adjusted odds (adjusted OR 3.28, 95% CI 1.72–6.25; *P* < .001). However, this association for CAC 1 to 99 was not found to be statistically significant after adjustment (adjusted OR 1.29, 95% CI 0.76–2.19; *P* = .344). The unadjusted and adjusted associations of each CAC category with impaired CFR are shown in Table 4; the adjusted estimates are obtained from the same multivariable model used for the covariate estimates shown in Table 5 (n = 467, 185 events).

Impaired CFR was defined as CFR < 2.0. *Adjusted for age, sex, BMI, hypertension, diabetes mellitus, dyslipidemia, smoking status, CKD, and abnormal myocardial perfusion. OR, CI. The adjusted estimates are from the same multivariable model shown in Table 5 (n = 467; 185 events).

### 3.7. Clinical and imaging predictors of impaired CFR

Multivariable analysis revealed that multiple demographic, clinical and imaging parameters were independently associated with impaired CFR. The adjusted odds of impaired CFR increased by 27% (adjusted OR 1.27, 95% CI 1.06–1.53; *P* = .010) with each 10-year increase in age and diabetes mellitus was also independently associated with impaired CFR (adjusted OR 1.71, 95% CI 1.10–2.66; *P* = .017). Compared with CAC = 0, CAC 100–399 and CAC ≥400 remained independently associated with impaired CFR, with adjusted ORs of 2.41 (95% CI 1.35–4.30; *P* = .003) and 3.28 (95% CI 1.72–6.25; *P* < .001), respectively, whereas CAC 1 to 99 was not (adjusted OR 1.29, 95% CI 0.76–2.19; *P* = .344). Impaired CFR was independently associated with abnormal myocardial perfusion (adjusted OR 2.14, 95% CI 1.37–3.35; *P* = .001). The complete univariable and multivariable results are reported in Table 5.

The dependent variable was impaired CFR (<2.0). The multivariable model (n = 467; 185 events) included all variables listed in the table, with CAC entered as a 4-level categorical variable (reference: CAC = 0). It is the same model as that presented in Table 4. OR = odds ratio; CI, CAC. CAC categories are compared with CAC = 0 (reference).

The multivariable analysis showed that age, diabetes mellitus, moderate to high CAC burden and abnormal myocardial perfusion were independently associated with impaired CFR. The strongest associations were observed for CAC ≥ 400 (adjusted OR 3.28, 95% CI 1.72–6.25) and CAC 100 to 399 (adjusted OR 2.41, 95% CI 1.35–4.30), while abnormal myocardial perfusion was also associated with increased odds of impaired CFR (adjusted OR 2.14, 95% CI 1.37–3.35). The variables independently associated with impaired CFR are shown in Figure 4 as adjusted ORs and 95% CIs.

**Figure 4.**
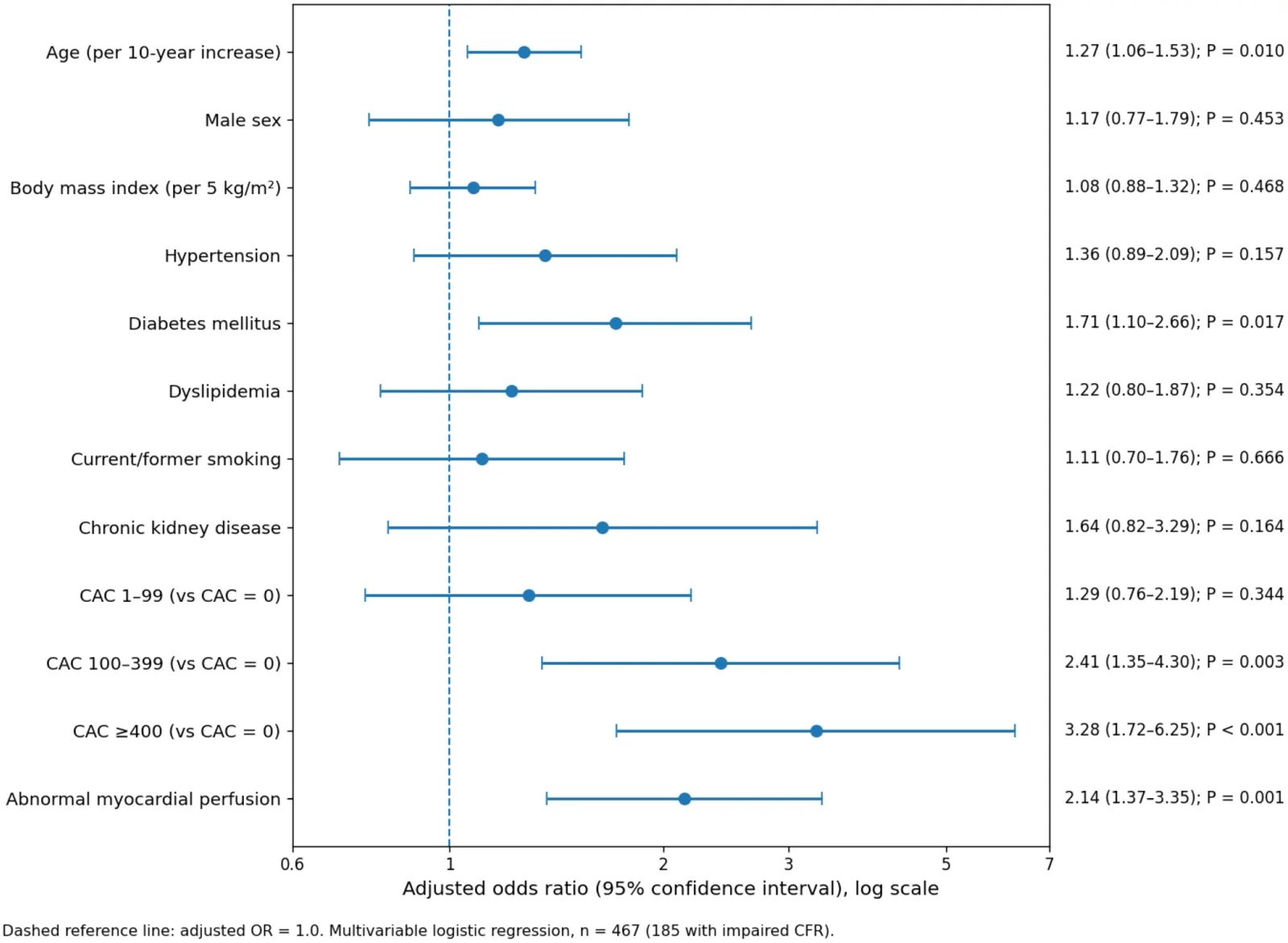
Variables independently associated with impaired coronary flow reserve.

Forest plot showing adjusted odds ratios and 95% CIs for demographic, clinical, and imaging variables associated with impaired CFR (CFR < 2.0). The vertical dashed line represents the null value (adjusted odds ratios = 1.0). Estimates positioned to the right of the reference line indicate an increased likelihood of impaired CFR. CAC. CAC 1 to 99, CAC 100 to 399 and CAC ≥ 400 are compared with CAC = 0 (reference). Estimates are from the multivariable model presented in Tables 4–5 (n = 467; 185 events); the x-axis is on a logarithmic scale.

### 3.8. CFR among patients with CAC = 0

However, out of the 193 patients with a CAC score of 0, 25.9% (50 patients) still had impaired CFR, suggesting that a low CFR was also present in the absence of calcium detection. Within this subgroup, patients with impaired CFR were older than those with preserved CFR (59.7 ± 11.6 vs 51.6 ± 13.4 years; *P* < .001) and had higher prevalences of hypertension (64.0% vs 36.4%; *P* < .001), diabetes mellitus (38.0% vs 14.0%; *P* < .001), and dyslipidemia (52.0% vs 31.5%; *P* = .009). Impaired CFR was also more common in patients with abnormal myocardial perfusion (36.0% vs 9.8%; *P* < .001). Stress MBF was substantially lower in the impaired CFR group (1.59 ± 0.42 vs 2.33 ± 0.59 mL/g/minutes; *P* < .001), resulting in mean CFR values of 1.66 ± 0.22 vs 2.70 ± 0.44 (*P* < .001). The results of this exploratory subgroup analysis are summarized in Table 6.

**Table 6:** Characteristics of patients with CAC = 0 according to coronary flow reserve status.

Variable	Preserved CFR ≥ 2.0 (n = 143)	Impaired CFR < 2.0 (n = 50)	*P* value
Age, yr	51.6 ± 13.4	59.7 ± 11.6	<.001
Male sex, n (%)	65 (45.5)	29 (58.0)	.128
Hypertension, n (%)	52 (36.4)	32 (64.0)	<.001
Diabetes mellitus, n (%)	20 (14.0)	19 (38.0)	<.001
Dyslipidemia, n (%)	45 (31.5)	26 (52.0)	.009
Current/former smoking, n (%)	24 (16.8)	13 (26.0)	.154
Abnormal myocardial perfusion, n (%)	14 (9.8)	18 (36.0)	<.001
Rest myocardial blood flow, mL/g/min	0.84 ± 0.18	0.99 ± 0.22	<.001
Stress myocardial blood flow, mL/g/min	2.33 ± 0.59	1.59 ± 0.42	<.001
CFR	2.70 ± 0.44	1.66 ± 0.22	<.001

This subgroup analysis included only patients with a CAC score of zero. CFR = CFR.

### 3.9. Sensitivity analyses

The inverse relationship between CAC burden and CFR held true across the sensitivity analysis as well as all of the exploratory subgroup analyses. After log transformation of CAC, it was independently associated with lower CFR (adjusted β = −0.21, 95% CI−0.28 to −0.14; *P* < .001) in continuous analysis. This relationship of CAC ≥ 400 and impaired CFR was also observed in patients with normal myocardial perfusion (n = 336; adjusted OR 2.74, 95% CI 1.28–5.87; *P* = .009). CFR was also significantly impaired in patients with moderate/high CAC (both with and without diabetes; adjusted OR 3.07 (95% CI 1.48–6.39); *P* = .003 and 2.19 (95% CI 1.28–3.74); *P* = .004, respectively). The inverse association between CAC burden and CFR was seen in both males and females. If a Bonferroni corrected threshold for the 7 analyses in Table 7 is used (*P* < .007), all associations, except for the association between patients with normal myocardial perfusion (*P* = .009) remained statistically significant. Due to the exploratory nature of the subgroup analyses, the results of these analyses are considered to be hypothesis generating. Table 7 presents the findings from these sensitivity and subgroup analyses.

Sensitivity analyses evaluated the consistency of the primary association using alternative CAC parameterization and clinically relevant subgroups. β = standardized regression coefficient; OR, CI. The continuous-CAC analysis was part of the planned analysis; the subgroup analyses by perfusion status, diabetes status and sex were exploratory, and their *P* values are not adjusted for multiple comparisons (Bonferroni corrected threshold for 7 analyses, *P* < .007).

## 4. Discussion

The study group included patients with suspected CAD, with a high prevalence of traditional cardiovascular risk factors such as aging, hypertension, diabetes mellitus, dyslipidaemia, obesity, and smoking. All these contribute to endothelial dysfunction, chronic inflammation, oxidative stress and vascular remodeling. The CFR may be reduced in such a population by a combination of various processes and the present data does not permit to assign the reduction to any single process. Quantitative MBF and CFR, however, do not only offer physiological data, but also data which goes beyond clinical evaluation, as in the joint position paper on behalf of the PET-derived MBF in the evaluation of suspected CAD it has been stated that this is the case.^[16]^ Likewise, multicenter trials have shown that compromised CFR is an independent risk factor for adverse cardiovascular outcomes in different patient populations.^[20]^

Higher CAC burden was associated with a worsening clinical profile, consistent with the hypothesis that CAC is an imaging marker of the cumulative burden of atherosclerotic risk factors. Persistent endothelial dysfunction, inflammation and osteogenic transformation of vascular smooth muscle cells likely drive vascular calcification, which is enhanced by aging, hypertension, diabetes mellitus, dyslipidemia, and CKD. These findings are consistent with the long-term data showing that the development of coronary calcification has a strong association with worsening cardiovascular risk factors.^[21]^ Likewise, higher CAC scores have been correlated with a higher risk of ischemic abnormalities on MPI, further substantiating its role as a marker of advanced coronary atherosclerosis.^[22]^

In all ascending categories of CAC, the capacity of coronary vasodilators was progressively less, and MBF was lower, suggesting that the higher the burden of calcified plaque the lower the coronary vasodilator capacity. Relatively preserved MBF at rest was defined by the presence of a small increase in higher CAC categories which could partially be due to higher blood pressure and older age; as a result, the CFR was lower mainly due to a blunted hyperemic response. Because CFR encompasses the epicardial and microvascular compartments, this pattern may reflect obstructive epicardial stenosis, diffuse nonobstructive atherosclerosis, endothelial dysfunction, coronary microvascular dysfunction, or a combination of these, and we cannot differentiate among them. A similar relationship between the severity of CAC and a decrease in MBF has been reported in patients with nonobstructive CAD.^[23]^ Furthermore, AI-based quantification of CAC using attenuation correction CT has shown excellent correlations with coronary flow capacity and prognosis, underscoring the importance of combining anatomical and functional imaging.^[24]^ However, CFR varied substantially within each CAC category, suggesting that calcified plaque accounts for only part of the variability in coronary vasodilator function.

After adjusting for these factors, demographically adjusted cardiovascular risk factors and myocardial perfusion results remained associated with impaired CFR, as did higher CAC categories. This suggests that CAC provides additional information on coronary vasodilator function beyond what traditional risk factors capture, but these findings do not necessarily imply that coronary calcification causes coronary vasodilator dysfunction; because the study is retrospective and cross-sectional, residual confounding is possible. The detection of reduced CFR in patients with lower CAC scores suggests that functional abnormalities may precede calcification or reflect coronary microvascular dysfunction. Similar results have been seen in trials showing that coronary CT and functional perfusion imaging offer complementary diagnostic information in detecting clinically significant ischemia.^[25]^ Similarly, coronary vascular abnormalities have been demonstrated even in the absence of obstructive coronary disease, underscoring the role of physiological assessment in addition to anatomical imaging.^[17]^

Factors associated with higher coronary calcium burden, such as diffuse atherosclerosis, endothelial dysfunction, and coronary microvascular impairment, can affect coronary blood flow measurements, including stress MBF and CFR. However, neither CAC nor reduced CFR is an accurate predictor of the specific epicardial anatomical substrate nor can it differentiate diffuse nonobstructive or focally obstructive plaque and microvascular dysfunction. Further definitive statements about relationship of calcium burden, plaque characteristics and impaired coronary flow would require high resolution intracoronary imaging, such as intravascular ultrasound (IVUS) and optical coherence tomography (OCT), which can directly assess the burden of calcium, dimensions, arc and thickness of the calcium, lesion morphology, and in the case of stents, related factors. Intravascular imaging, in addition to angiography, has been demonstrated to be clinically important by a meta-analysis of the long-term results of IVUS- and OCT-guided versus angiography-guided stent implantation.^[26]^ Future studies using quantitative CFR and IVUS/OCT-derived evaluation of coronary flow are therefore warranted to more precisely identify the anatomical substrate of impaired coronary flow in patients with a high CAC burden.

The association of age, diabetes mellitus and abnormal myocardial perfusion with impaired CFR also supports the notion that coronary vasodilator function is a composite of aging and metabolic risk. CFR can therefore be considered a whole-vessel assessment of coronary vascular health, not just epicardial stenosis alone. These findings agree with a study showing that CFR has better prognostic significance in patients without obstructive CAD.^[27]^ Moreover, temporal changes in CFR have been associated with subsequent cardiovascular outcomes.^[28]^

Notably, 25.9% of patients with CAC = 0 had impaired CFR, indicating that coronary vasodilator dysfunction can occur in the absence of detectable calcified plaque. Several mechanisms have been suggested to explain low CAC and impaired CFR, including coronary microvascular dysfunction, endothelial impairment, diffuse noncalcified atherosclerosis, and metabolic abnormalities, although these were not directly assessed in the present study. Physiological assessment may thus be used to identify the low risk patient, who would not have been classified as low risk based on CAC alone. Impaired myocardial perfusion has been shown to precede clinical CAD, particularly in individuals with hypertension and metabolic disorders^[29]^ and age- and sex-adjusted reference values indicate that a relatively low anatomical level of disease is sufficient to cause physiological impairment.^[30]^ Patients who are symptomatic with CAC = 0 may also have reduced CFR and adverse cardiovascular outcomes^[11]^ while a CAC category might be used to exclude significant ischemia at the population level but not in all patients with reduced CFR and functional coronary abnormalities.^[31]^

The results of the sensitivity analyses showed that the relationship between coronary artery calcium burden and CFR was persistent and did not appear to be due to the specific analytical models or to single covariates. However, because the subgroup analyses were exploratory and not adjusted for multiple comparisons, they should be interpreted with caution. These findings are in keeping with recent findings that quantitative CAC measurement adds diagnostic value to PET MPI beyond established clinical variables.^[32]^ Similarly, when observing a severely reduced CFR, the recognized interindividual variability highlights the need to carry out sensitivity analyses to verify that the observed association reflects real myocardial impairment and not analytical variation.^[33]^

### 4.1. Strengths of the study

One of the strengths of this study is the combination of assessment of anatomical coronary atherosclerotic burden and coronary vasodilator function in a relatively large patient population of 467 patients who met criteria for suspected CAD. The study assessed the relationship between structural coronary calcification and functional coronary abnormalities, using quantitative MBF measurements as well as CFR, going beyond coronary calcium burden. Defined CAC categories (0, 1–99, 100–399, and ≥400), a clinically relevant CFR threshold of <2.0, quantitative perfusion imaging and multivariable adjustment for demographic, cardiovascular, and imaging-related variables supported independent associations. The dose-dependent decrease of CFR across different categories of CAC burden and inverse correlation between the continuous burden of CAC and CFR were consistent across all complementary analyses. Importantly, the correlation between high CAC burden and impaired CFR remained after adjustment and was consistent in the sensitivity analysis and in exploratory subgroup analyses by sex and diabetes status. These features support the strength and significance of the observed anatomical–functional relationship.

### 4.2. Limitations

Some restrictions need to be taken into account when interpreting these findings. First, the design of this retrospective observational study precludes causal inference, and the patients included may not be representative of asymptomatic patients or the general population, which may introduce selection bias. Secondly, the analysis was based on data routinely collected from clinical and imaging data, and some information may have been lost or differed across the various imaging acquisitions and reporting. While technically, unreliable examinations and records with substantial missing data were not included, there may be residual confounding due to unmeasured variables such as the severity of diffuse coronary atherosclerosis or microvascular dysfunction. Furthermore, CFR is a measure of epicardial and microvascular coronary function, and cannot thus differentiate the underlying mechanism of decreased coronary vasodilator capacity. Ultimately, the study was carried out in a tertiary cardiovascular center and was a retrospective study over a defined period of time, which may restrict the applicability of the results to other healthcare environments and populations. Furthermore, no a priori sample size calculation was done and the exploratory subgroup analyses were not adjusted for multiple comparisons, thus, type I error cannot be ruled out. The absolute MBF measurements are dependent on the imaging modality, the tracer, the reconstruction, the software and the kinetic model and cannot be directly translated to other platforms. While a small number of adolescents (16–17 years) were included, this may reduce the generalizability of the findings to the pediatric population. Furthermore, invasive (or CT) coronary angiographic information was not systematically available, and the severity and presence of obstructive epicardial stenosis could not be fully accounted for, and no intravascular imaging (IVUS or OCT) was performed, which meant that plaque burden and plaque morphology were unavailable for direct assessment. To further validate these associations, prospective, multicentre studies with standardized quantitative flow assessment and prospective clinical outcomes are warranted.

## 5. Conclusion

The present study, using a retrospective analysis in patients with suspected CAD who had undergone QMPI, revealed a graded and inverse relationship between coronary artery calcium burden and CFR. The prevalence of impaired CFR increased from 25.9% to 67.1%, and the mean value of CFR progressively decreased from 2.43 ± 0.62 in patients with no detectable coronary calcium to 1.57 ± 0.47 in patients with CAC ≥ 400. After adjusting for demographic characteristics, cardiovascular risk factors and myocardial perfusion findings, the association of higher CAC burden with impaired CFR remained independent. Other factors such as age and diabetes mellitus and abnormal myocardial perfusion were also independently associated with impaired CFR. Importantly, this relationship between high CAC burden and impaired CFR was seen in patients with normal myocardial perfusion (exploratory analysis), which could help identify those in whom quantitative flow assessment may demonstrate abnormal vasodilator function that would not be apparent by conventional perfusion assessment. These results reflect associations, and cannot be used to infer that coronary calcium is causative of coronary physiological impairment, due to the retrospective, cross-sectional study design. In summary, these results provide further evidence that combining coronary calcium burden and quantitative CFR is a valuable tool for assessing patients with suspected CAD. Future prospective and multicentre studies are required to elucidate whether anatomical and functional evaluation results in better risk stratification and to predict future cardiovascular events.

## Author contributions

**Methodology:** Lingling Zhang.

**Writing – original draft:** Lingling Zhang.

**Conceptualization:** Fan Wu.

**Project administration:** Fan Wu.

**Validation:** Fan Wu.
